# Successful treatment of mucosal neuromas by radiofrequency ablation in a patient with multiple endocrine neoplasia type 2B

**DOI:** 10.1002/ski2.146

**Published:** 2022-09-28

**Authors:** Luis Escalante, Jennyfer Granizo‐Rubio, Esteban Ortiz‐Prado, Víctor Pinos‐León, Astrid Maldonado, David Chandler

**Affiliations:** ^1^ Dermatology Unit Central University of Ecuador Quito Ecuador; ^2^ EPHORA Research Group Guayaquil Ecuador; ^3^ Dermatology Department Eugenio Espejo Hospital Quito Ecuador; ^4^ One Health Research Group De las Americas University Quito Ecuador; ^5^ Department of Cell Biology, Physiology and Immunology University of Barcelona Barcelona Spain; ^6^ DermaAID Centro Dermatológico de Alta Especialidad Quito Ecuador; ^7^ Dermatology Department University Hospitals Sussex NHS Foundation Trust Worthing UK; ^8^ Department of Global Health & Infection Brighton and Sussex Medical School Brighton UK

## Abstract

This is the first report of mucosal neuromas being treated successfully with radiofrequency ablation in a patient with multiple endocrine neoplasia type 2B.

## Graphical abstract

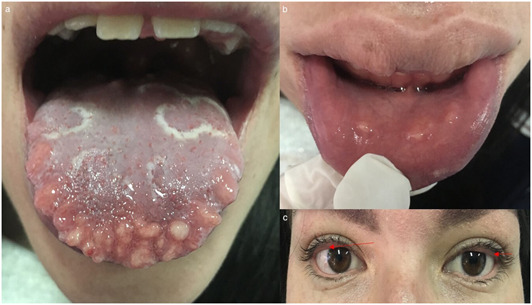

1

**What's already known about this topic?**

- Mucosal neuromas are very common in patients with MEN2B
- They can be highly symptomatic and interfere with everyday activities such as eating and speaking
- Evidence on effective treatment options for mucosal neuromas is lacking

**What does this study add?**

- We describe the first case of RFA being used to treat mucosal neuromas in a patient with MEN2B
- Treatment resulted in rapid symptomatic and aesthetic improvement
- The procedure is straightforward and can be performed in the consultation room

## 2 CASE SUMMARY

A 24‐year‐old female presented to our department with a 10 years history of progressive lip enlargement, with numerous lumps on the tongue and lips. The lumps were painful and interfered with eating and speech, frequently becoming traumatized. Her medical history included medullary thyroid cancer with lymphatic and pulmonary metastases, for which she had undergone total thyroidectomy with lymph node resection and treatment with a tyrosine kinase inhibitor (sorafenib).

**FIGURE 1:**
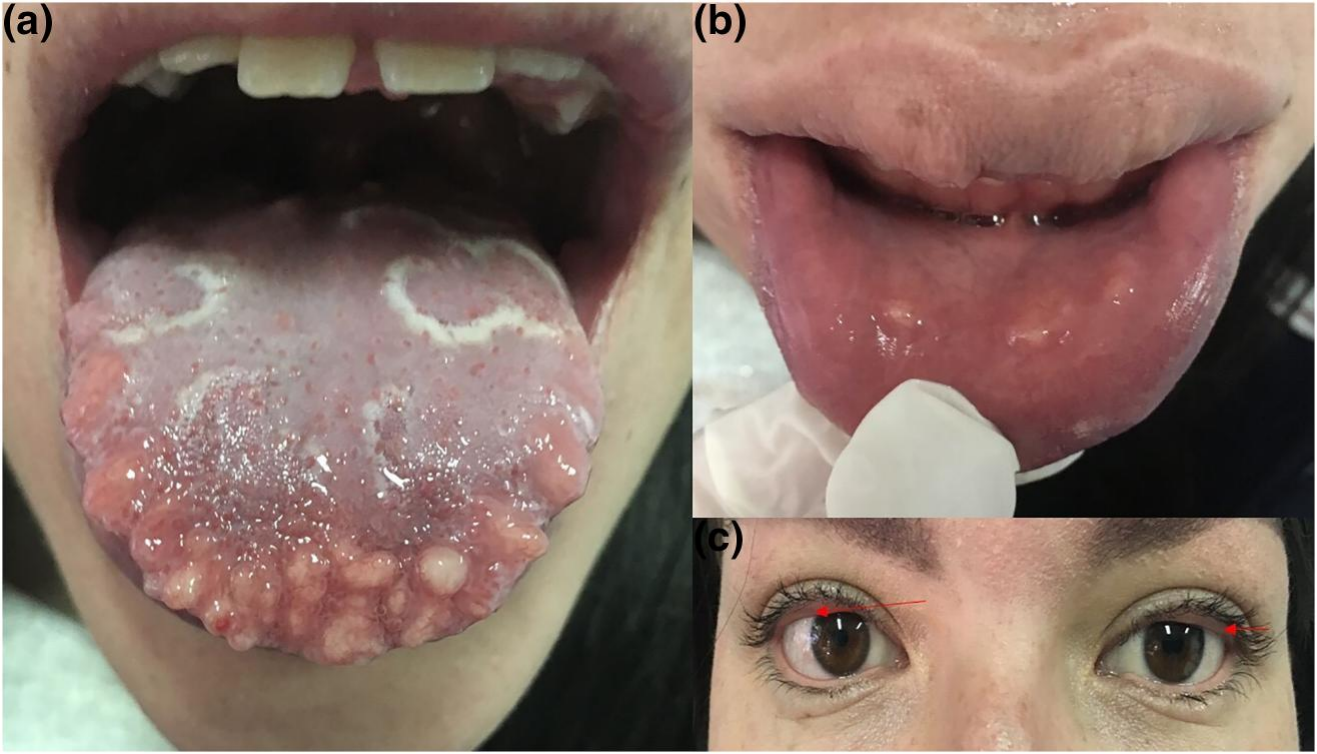
(a) and (b) Multiple smooth‐shiny yellow papules on the surface on the tongue and lower lip. Geographic tongue is also present. (c). Yellow papules on the upper palpebral conjunctiva bilaterally (red arrows) with associated eyelid eversion

**FIGURE 2:**
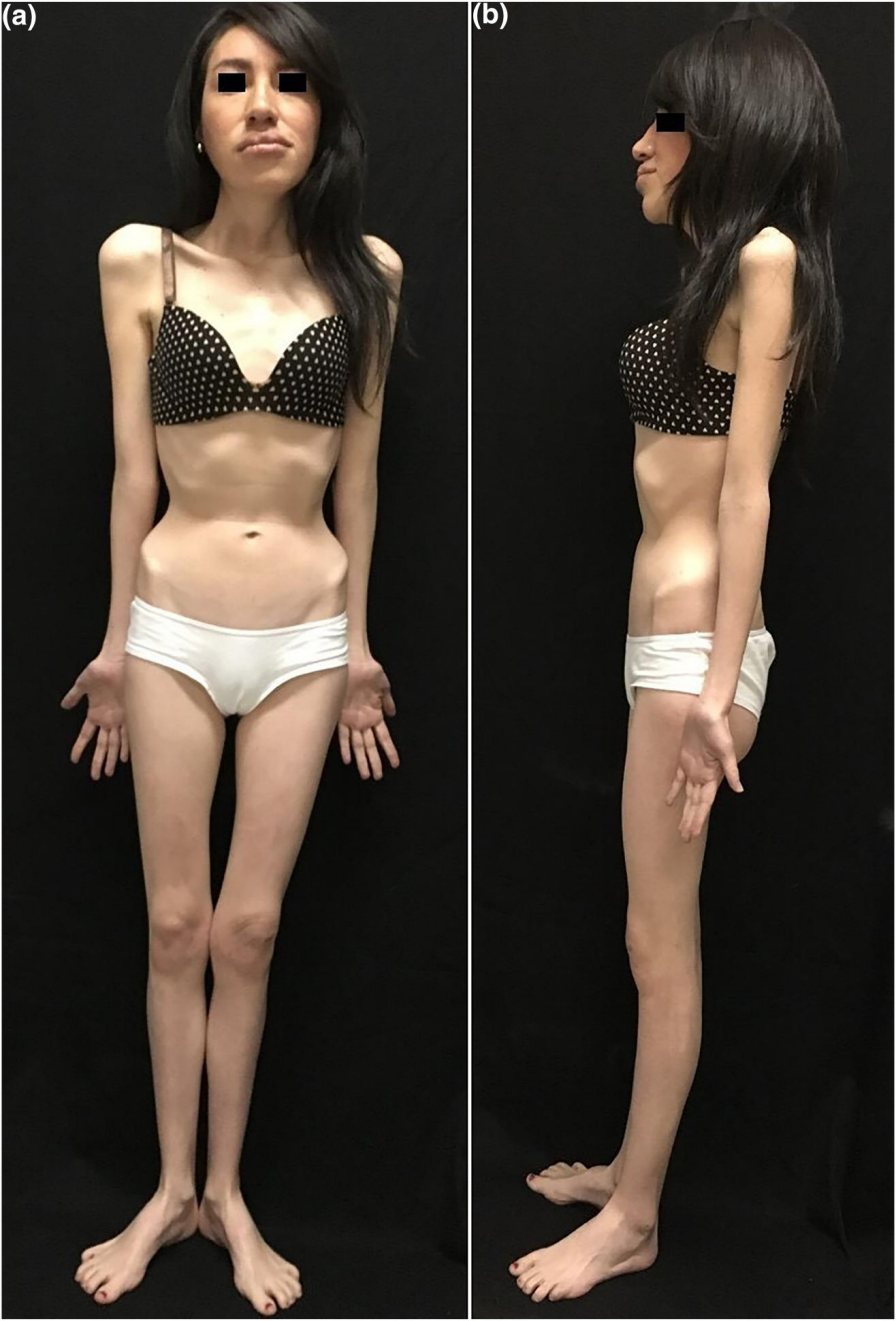
(a) and (b) Marfanoid habitus, dolichocephaly, above‐average height, long extremities and prognathism

**FIGURE 3:**
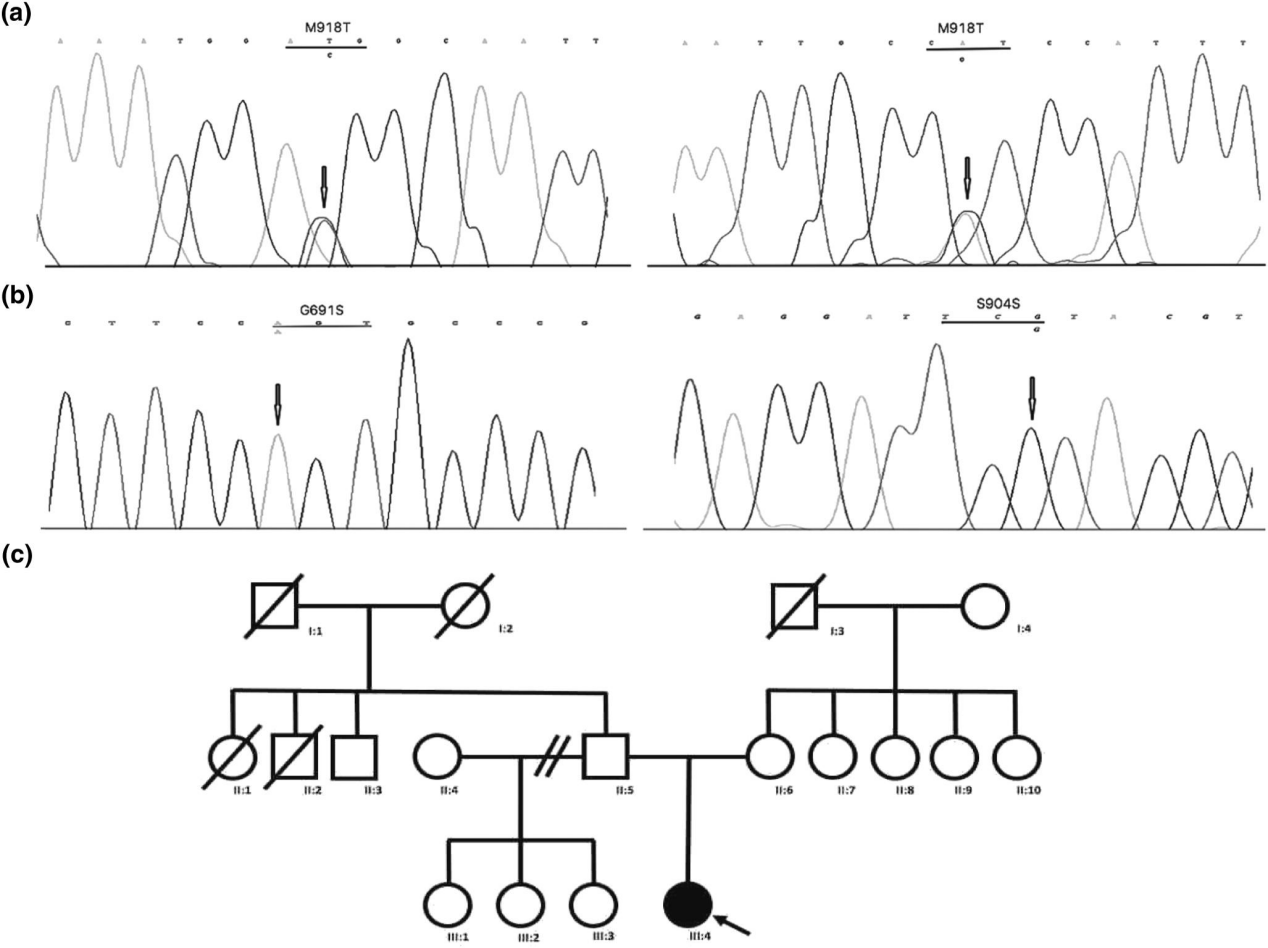
(a) pM918T heterozygous point mutation (exon 16), sense and anti‐sense sequence. (b) Polymorphisms G961S (exon 11) and S904S (exon 15) shown in the left and right panels respectively. (c) De novo mutation shown in the patient's family tree

Examination revealed multiple smooth yellow papules involving the tongue, labial mucosae and the upper palpebral conjunctivae (Figure 1). Of note, the patient also exhibited a marfanoid habitus, with dolichocephaly and prognathism (Figure 2). Biopsy of eyelid and tongue lesions confirmed that these were mucosal neuromas, and subsequent genetic testing confirmed the diagnosis of multiple endocrine neoplasia type 2B (MEN2B) identifying de novo point mutations in the RET proto‐oncogene (M918T, G691S, S904S) (Figure 3).

**FIGURE 4:**
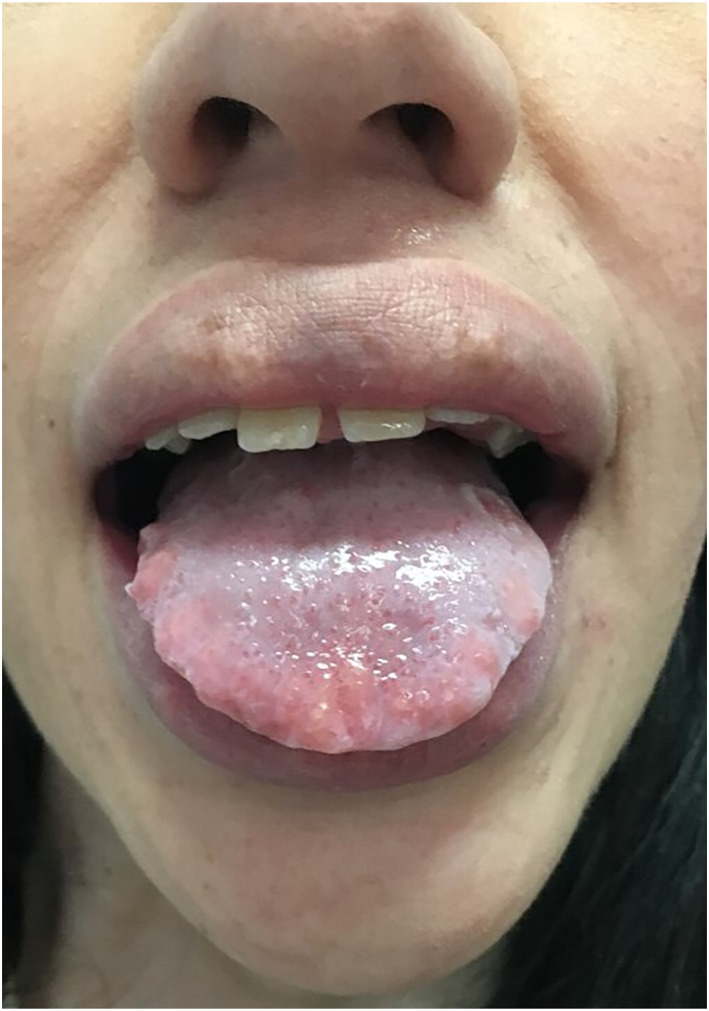
Normal appearance of the tongue 6 months after radiofrequency ablation

The lingual neuromas were treated by radiofrequency ablation (RFA) applied at 500 kHz. Each neuroma was treated once per session with a continuous pulse lasting 5–10 s, and the patient underwent three treatment sessions in total. Local anaesthesia was achieved by injection of 2% lidocaine with 1:200 000 adrenaline. Postoperative management included pain control with paracetamol and topical anaesthetic (benzocaine 20% gel); antibiotic prophylaxis was not given. The patient made a quick recovery, and reported complete resolution of symptoms. At follow‐up review 6 months after surgery the patient remained pain‐free and satisfied with the aesthetic outcome (Figure 4).

## 3 DISCUSSION

MEN2B is a rare autosomal dominant condition caused by mutations in the RET proto‐oncogene. A point mutation in the tyrosine kinase domain of the RET gene at codon 918 (exon 16) is identified in 95% of individuals resulting in the substitution of threonine for methionine (M918T). The other mutations identified in our patient, in exon 11 (G691S, rs1799939, GGT > AGT, Gly > Ser) and exon 15 (S904S, rs1800863, TCC > TCG, Ser > Ser) are present in the homozygous state and have been described as polymorphic variants without clinical association.

Clinically MEN2B is characterized by medullary thyroid cancer, phaeochromocytoma, and developmental abnormalities including marfanoid habitus. Mucosal neuromas are a common feature and can be troublesome for patients, yet there is no guidance on how best to manage this particular problem. Mucosal neuromas in MEN2B are found on the lips, conjunctivae and tongue, and in the intestines. Progressive growth of conjunctival neuromas can cause eyelid eversion, generating a characteristic feature of this syndrome. Neuromas on the tongue are commonly observed on the tip or lateral borders. Geographic tongue was an additional feature in our patient (Figure 1) which has not been reported in association with MEN2B. Histologically, mucosal neuromas are characterized by irregular and tortuous bundles of nerve fibres with a thickened perineum within the submucosa. Plexiform neurofibromas may have a similar histological appearance, although immunohistochemical markers can distinguish these two entities.

Evidence on effective treatment for mucosal neuromas is limited, with one case report describing a favourable response to CO_2_ laser.^1^ Surgical excision is an option, however lesions can recur after treatment.^2^

To our knowledge this is the first report of mucosal neuromas treated successfully by RFA. In our experience, the procedure was easy to perform and resulted in excellent clinical outcome with no complications. RFA is an established treatment for symptomatic interdigital (Morton's) neuroma, but has also been used to treat oral pathologies including mucocoeles, lymphangiomas and lymphatic malformations, viral warts and malignancies such as rhabdomyosarcoma. Furthermore, the treatment of oral pathologies using RFA has been well‐tolerated in the paediatric population.^3^, ^4^ RFA can be used in high and low‐frequency modes. High‐frequency ablation is useful for destruction of deep (submucosal) tissue causing a reduction in tissue volume due to fibrosis. In low‐frequency mode, accurate destruction of superficial (mucosal) tissue can be achieved at low‐temperature, causing minimal thermal injury to surrounding tissue and allowing the patient to make a speedier recovery.

Although the procedure is relatively straightforward to perform, care is required to remove only the neuromas without affecting the lingual shape (edge). The triangular tip of the radiofrequency probe is invaluable when performing this procedure, and the operator must ensure a smooth and continuous movement to obtain favourable aesthetic results. The procedure is made challenging by the continuous movement of the tongue, and a foerster clamp can be useful to hold the tongue in a steady position and provide optimal exposure of the neuromas. The risk of oedema, bleeding and infection is low, although the latter will require the addition of systemic antibiotics. A soft diet after the treatment is essential to maintain adequate epithelialisation of the treated areas.

Despite the distinctive physical characteristics usually reported in patients affected by MEN2B, diagnosis can be delayed especially in settings where genetic testing is not available. A wide range of skin manifestations can occur in association with inherited cancer syndromes such as MEN2B, and these are well described in children and adults.^5^, ^6^ Dermatologists can assist in the management of patients with MEN2B by recognizing these features, and providing treatment for mucosal neuromas.

We propose that radiofrequency ablation should be considered as a treatment option for patients of any age with symptomatic mucosal neuromas, including patients with MEN2B and those with solitary mucosal neuromas. RFA may also offer an effective treatment option for patients with other neurogenic tumours in the oral cavity including neurofibroma, neurilemmoma and Palisaded encapsulated neuroma (PEN).

## AUTHOR CONTRIBUTION

**Luis Escalante**: Conceptualization (lead); Investigation (equal); Writing – original draft (lead); Writing – review & editing (supporting). **Jennyfer Granizo‐Rubio**: Investigation (equal). **Esteban Ortiz‐Prado**: Writing – original draft (supporting). **Victor Pinos León**: Investigation (equal). **Astrid Maldonado**: Investigation (equal); Writing – original draft (supporting). **David Chandler**: Conceptualization (supporting); Writing – original draft (lead); Writing – review & editing (lead).

## CONFLICT OF INTEREST

The authors declare that there is no conflict of interest that could be perceived as prejudicing the impartiality of the research reported.

## ETHICS STATEMENT

Written, informed consent was provided by the patient. IRB approval is not required.

## Data Availability

Further data available on request from the authors.
